# Y-chromosome polymorphisms and ethnic group – a combined STR and SNP approach in a population sample from northern Italy

**DOI:** 10.3325/cmj.2013.54.279

**Published:** 2013-06

**Authors:** Venusia Cortellini, Andrea Verzeletti, Nicoletta Cerri, Alberto Marino, Francesco De Ferrari

**Affiliations:** 1 Department of Forensic Medicine, University of Brescia, Brescia, Italy; 2 Carabinieri Corp Forensic Department, Parma, Italy

## Abstract

**Aim:**

To find an association between Y chromosome polymorphisms and some ethnic groups.

**Methods:**

Short tandem repeats (STR) and single-nucleotide polymorphisms (SNP) on the Y chromosome were typed in 311 unrelated men from four different ethnic groups – Italians from northern Italy, Albanians, Africans from the Maghreb region, and Indo-Pakistanis, using the AmpFlSTR^®^ Yfiler PCR Amplification Kit and the SNaPshot Multiplex Kit.

**Results:**

STRs analysis found 299 different haplotypes and SNPs analysis 11 different haplogroups. Haplotypes and haplogroups were analyzed and compared between different ethnic groups. Significant differences were found among all the population groups, except between Italians and Indo-Pakistanis and between Albanians and Indo-Pakistanis.

**Conclusions:**

Typing both STRs and SNPs on the Y chromosome could become useful in determining ethnic origin of a potential suspect.

Determining the ethnic origin of a suspect through DNA analysis of biological stains left at the crime scene is an important part of criminal investigations. To discriminate between different ethnic groups, short tandem repeat (STR) autosomal marker analysis (1-6) can be complemented by single-nucleotide polymorphism (SNP) assays, which have have been demonstrated to be more useful for this purpose (7,8). The introduction of new markers, mostly from the Y chromosome, offers a better power of discrimination to define even sub-populations of different ethnic groups (9-11). This study aims to compare a sample of Italian men from Brescia (northern Italy) with a sample of men from each of three main ethnic groups living in Brescia county (Albanians, North Africans, Indo-Pakistanis), through STRs and SNPs Y chromosome typing, in order to find the data useful in defining the ethnic origin.

## Materials and methods

The study was conducted on samples collected during routine forensic work of the Department of Forensic Medicine of Brescia, Italy, over the period between 2010 and 2012. A total of 311 men from four different ethnic groups were evaluated: 107 Italians, 83 Albanians, 77 Africans from Maghreb, and 44 Indo-Pakistanis. The participants were not related and the samples were of sufficient quality and quantity to be included in the statistical analysis.

DNA was extracted from buccal swabs or blood using the Chelex^®^ 100 procedure (12). All the samples were genotyped for 17 Y-chromosome STRs using the AmpFlSTR^®^ Yfiler PCR Amplification Kit (Applied Biosystems, Foster City, CA, USA), which allows co-amplification of the core set of the European Minimal Haplotype (DYS389I, DYS390, DYS389II, DYS19, DYS385 a/b, DYS393, DYS391, DYS439, DYS635, DYS392) and seven other loci (DYS437, DYS438, DYS448, DYS456, DYS458, DYS635, Y GATA H4), according to manufacturer’s recommendations. The amplification was carried out in a GeneAmp^®^ PCR System 9700 Gold Plate (Applied Biosystems).

Samples were also analyzed for 18 SNPs (M170, M172, M35, M9, M45, M173, M89, M267, M282, M304, M214, M52, M201, M96, M181, M174, M91, M216), belonging to the non-recombinant region of Y chromosome, through two multiplexes, arbitrarily called MY1 and MY2, containing 10 and 8 markers respectively, by SNaPshot Multiplex Kit (Applied Biosystems) (13-15).

STR and SNP typing was performed in an ABI Prism^®^ 310 Genetic Analyzer (Applied Biosystems); STRs allele calling was performed through GeneMapper ID^®^ v3.2 software, using manufacturer’s allelic ladders, bins, and panels. For SNPs calling, bins and panels were manually defined in GeneMapper ID^®^ v3.2 software.

Allele, haplotype, and haplogroup frequencies were estimated by direct counting. Haplogroup was defined according to YCC nomenclature (16,17). Haplotypes of the four population groups were compared using the ARLEQUIN Software, version 3.1 (18): the variance of allele frequencies between populations and the probability of identity by descent was calculated (F_ST_ – Fixation Index Statistics).

## Results

STRs analysis found 299 different haplotypes out of 311 samples (107/107 Italians, 81/83 Albanians, 68/77 Africans, 43/44 Indo-Pakistanis): allelic frequencies inside the four ethnic groups are reported respectively in Table 1, Table 2, Table 3, and Table 4. Haplotypes of the four population groups were compared using the ARLEQUIN Software (18) (Table 5).

**Table 1:** Allele frequencies for 17 Y-short tandem repeat loci in a population sample from northern Italy (N = 107)*

Alleles	Italians																Allelic class
	DYS456	DYS389I	DYS390	DYS389II	DYS458	DYS19	DYS393	DYS391	DYS439	DYS635	DYS392	Y GATA H4	DYS437	DYS438	DYS448	DYS 385 a/b	
8												0.0093				0.0093	10-13
9								0.0187						0.0748		0.0093	10-14
10								**0.5234**	0.0467			0.0093		**0.5514**		0.0093	10-17
11							0.0093	0.4579	0.2056		0.3645	**0.5514**		0.0374		0.0093	11-12
12		0.2056					0.1121	0.0280	**0.5887**		0.0280	0.4299		0.3177		0.0654	11-13
13	0.0467	**0.7009**				0.1215	**0.7383**		0.1215		**0.5794**	0.0280		0.0467		**0.3645**	11-14
14	0.0841	0.1121			0.0187	**0.5888**	0.1495		0.0373		0.0467		0.2430			0.0561	11-15
15	**0.4673**	0.0093			0.1402	0.2523	0.0093				0.0093		**0.5888**			0.0093	11-16
16	0.3271				0.2430	0.0280							0.1963			0.0187	11-17
17	0.0654				**0.3458**	0.0280									0.0093	0.0093	12-12
18	0.0093				0.2056	0.0093									0.0374	0.0093	12-13
18.2					0.0093											0.0748	12-14
19					0.0561					0.0093					**0.5514**	0.0187	12-15
20					0.0093					0.0374					0.3084	0.0093	12-18
21			0.0280							0.2149					0.0654	0.0654	13-14
22			0.0654							0.0934					0.0467	0.0374	13-15
23			0.2991							**0.5140**					0.0093	0.0093	13-16
24			**0.5607**							0.1308						0.0093	13-17
25			0.0654							0.0280						0.0093	13-18
26			0.0093													0.0374	14-14
27				0.0093												0.0187	14-15
28				0.1121												0.0093	14-16
29				**0.5794**												0.0187	14-17
30				0.2430												0.0093	14-18
31				0.0467												0.0187	15-16
32				0.0280												0.0093	15-17
33				0.0093												0.0093	15-18
																0.0093	16-16
																0.0093	16-17
																0.0280	16-18
																0.0280	17-18
																0.0093	17-19

*In bold, the most frequent allele.

**Table 2:** Allele frequencies for 17 Y-short tandem repeat loci in a population sample from Albania living in northern Italy (N = 83)*

Alleles	Albanians																Allelic class
	DYS456	DYS389I	DYS390	DYS389II	DYS458	DYS19	DYS393	DYS391	DYS439	DYS635	DYS392	Y GATA H4	DYS437	DYS438	DYS448	DYS385 a/b	
8														0.0120		0.0120	10-10
9								0.0120	0.0120		0.0241			0.2290		0.0120	10-11
10								**0.7470**	0.0361		0.0361	0.0241		**0.5181**		0.0120	10-14
11								0.2290	0.2892		**0.7953**	**0.5182**		0.1084		0.0724	11-11
12		0.1808				0.0120	0.2772	0.0120	**0.5422**		0.0361	0.3976		0.1325		0.0844	11-14
13	0.1446	**0.7348**				**0.3373**	**0.6385**		0.1205		0.0964	0.0602	0.0120			0.0120	11-15
14	0.0723	0.0843			0.0602	0.2772	0.0602						**0.5061**			0.0120	12-14
15	**0.3253**				**0.2772**	0.2169	0.0241				0.0120		0.2771			0.0120	12-16
16	0.2650				**0.2772**	0.1446							0.2048			0.0120	12-18
17	0.1928				0.1928	0.0120										0.0120	13-14
17.2					0.0120											0.0120	13-15
18					0.1084										0.0361	0.0362	13-17
18.2					0.0120											0.0120	13-18
19					0.0120										0.3254	0.0120	13-19
20					0.0482					0.0602					**0.5301**	0.0120	13-20
21			0.0120							0.2651					0.0723	0.0120	14-14
22			0.0723							0.3012					0.0361	0.1085	14-15
23			0.2048							**0.3253**						0.0362	14-16
24			**0.5423**							0.0362						0.0965	14-17
25			0.1566													0.0241	14-18
26			0.0120							0.0120						0.0120	14-19
27				0.0120												0.0120	15-17
28				0.1325												0.0120	15-17.1
29				0.2892												0.0483	15-18
30				**0.4218**												0.0120	15-19
31				0.1325												0.0120	15.3-18
32				0.0120												0.0241	16-16
																0.0362	16-17
																0.0120	16-17.3
																**0.1206**	16-18
																0.0362	16-19
																0.0603	17-18

*In bold, the most frequent allele.

**Table 3:** Allele frequencies for 17 Y- short tandem repeat loci in a population sample from the Maghreb region living in northern Italy (N = 77)*

Alleles	North Africans																Allelic class
	DYS456	DYS389I	DYS390	DYS389II	DYS458	DYS19	DYS393	DYS391	DYS439	DYS635	DYS392	Y GATA H4	DYS437	DYS438	DYS448	DYS 385 a/b	
9								0.2727	0.0130					0.0909		0.0130	9-11
10							0.0390	**0.5065**	**0.4286**		0.0519	0.0650		**0.8312**		0.0130	11-13.2
11		0.0130					0.0130	0.1948	0.3376		**0.8443**	**0.5065**		0.0130		0.0130	11-20
12		0.2857					0.2987	0.0260	0.1688			0.3766		0.0649		0.0130	12-13
13		0.2597				**0.5195**	**0.6103**		0.0390		0.0519	0.0519				0.0130	12-14
14	0.1558	**0.4156**			**0.2208**	0.3117	0.0260				0.0519		**0.8312**			0.0130	12-15
15	0.2468	0.0260			0.0650	0.1298	0.0130		0.0130				0.1169			0.0130	12-18
16	**0.5714**				0.1688	0.0130							0.0519			**0.2078**	13-14
16.2					0.0130											0.1299	13-15
17	0.0260				0.1948	0.0260										0.0259	13-16
18					0.1558										0.0519	0.0259	13-17
18.2					0.0909											0.0130	13-18
19					0.0519										0.1429	0.1039	13-19
19.2					0.0390											0.0130	13-20
20										0.0779					**0.7013**	0.0259	14-14
21			0.0130							**0.7143**					0.1039	0.0390	14-15
22			0.0910							0.0909						0.0390	14-16
23			**0.4545**							0.0779						0.0259	14-19
24			0.4287							0.0390						0.0130	15-17
25			0.0130													0.0259	16-17
28				0.0390												0.0130	16-18
29				**0.3766**												0.0130	17-17
30				**0.3766**												0.1428	17-18
31				0.1429												0.0390	17-19
32				0.0519												0.0130	18-19
33				0.0130													

*In bold, the most frequent allele.

**Table 4:** Allele frequencies for 17 Y- short tandem repeat loci in a population sample from India and Pakistan living in northern Italy (N = 44)*

Alleles	Indo-Pakistanis																Allelic class
	DYS456	DYS389I	DYS390	DYS389II	DYS458	DYS19	DYS393	DYS391	DYS439	DYS635	DYS392	Y GATA H4	DYS437	DYS438	DYS448	DYS 385 a/b	
9														0.2955		0.0227	9-15
10								**0.7273**	0.3183		0.1137			0.2273		0.0227	9-16
11							0.0455	0.2500	**0.3635**		**0.6818**	0.2955		**0.4318**		**0.2955**	11-14
12		0.1591					0.2727	0.0227	0.2273		0.0682	**0.5227**		0.0454		0.0682	11-15
13	0.0909	**0.5909**				0.0682	**0.6136**		0.0909		0.0454	0.1818				0.0682	12-13
14	0.0909	0.2500			0.0227	0.2954	0.0682				0.0909		**0.6818**			0.0455	12-14
15	**0.5000**				0.1818	**0.3409**							0.1818			0.0227	12-15
16	0.2500				**0.3182**	0.2500							0.1364			0.0227	13-14
17	0.0682				0.2273	0.0455										0.0682	13-17
18					0.1591										0.0227	0.0455	13-18
19					0.0682										0.4091	0.0227	13-19
19.2					0.0227											0.0227	13-20
20										0.1137					**0.5455**	0.0455	14-17
21			0.0227							0.2500					0.0227	0.0227	14-18
22			0.2045							0.0227						0.0227	15-16
23			0.2045							**0.3864**						0.0682	15-17
24			**0.3183**							0.1818						0.0227	15-17.1
25			0.2500							0.0227						0.0682	15-18
26										0.0227						0.0227	16-20
27				0.0909													
28				0.0682													
29				0.2273													
30				**0.3863**													
31				0.1364													
32				0.0682													
33				0.0227													

*In bold, the most frequent allele.

**Table 5:** Haplotype comparison among four different population samples (Italians, Albanians, North Africans, Indo-Pakistanis): pairwise F_ST_ * (Fixation Index Statistics)

Populations	Italians	Albanians	North Africans
Albanians	**0.03223 ± 0.0056**		
North Africans	**0.00000 ± 0.0000**	**0.00000 ± 0.0000**	
Indo-Pakistanis	0.99902 ± 0.0002	0.30762 ± 0.0162	**0.02930 ± 0.0068**

*F_ST_ *P* – value of population comparison. Number of permutations: 10 000. In bold – significant differences (*P* < 0.05).

No significant differences were found between Italians and Indo-Pakistanis, as opposed to Italians and Albanians, between whom significant differences were found, as well as between Italians and North Africans. Significant differences were found between North Africans’ haplotypes and all other populations.

SNPs analysis showed 11 different haplogroups, the most represented being E1b1b1 (28.0%), J2 (10.9%), and R1 (31.5%) (Table 6). Among 107 Italian men, 7 different haplogroups were found, the most frequent being R1 (60.75%). Among 83 men from Albania, 9 different haplogroups were found, the most frequent being E1b1b1 (32.53%). Among 77 men from North Africa, 8 different haplogroups were found and the most frequent was E1b1b1 (57.14%). Among 44 Indo-Pakistani men, 8 different haplogroups were found and the most frequent was R1 (38.64%). According to the haplogroup frequency distribution, the four populations can be grouped into two main clades: Italians/Indo-Pakistanis and Albanians/North Africans.

**Table 6:** Haplogroup frequencies in four different population samples (Italians, Albanians, North Africans, Indo-Pakistanis)*

Haplogroups	Italians	Albanians	North Africans	Indo-Pakistanis	Total
E1b1b1	0.1100	0.3250*	**0.5710**	0.0910	0.2790
F	0.0000	0.0120	0.0130	0.0000	0.0060
G	0.1100	0.0000	0.0000	0.0000	0.0390
H1	0.0000	0.0240	0.0000	0.1590	0.0290
I	0.0610	0.1210	0.0130	0.0230	0.0610
J1	0.0090	0.0480	0.1950	0.0460	0.0710
J2	0.0654	0.1687	0.1040	0.1140	0.1090
K*(xNOP)	0.0190	0.1450	0.0260	0.0910	0.0640
NO	0.0000	0.0000	0.0130	0.0000	0.0030
P*(xR1)	0.0090	0.0240	0.0000	0.0910	0.0230
R1	**0.6080**	0.1330	0.0650	**0.3860**	0.3150

*In bold, the most frequent haplogroup.

## Discussion

The obtained data confirm the high variability of Y-STRs both within and among populations. This situation indicates a very weak genetic structure in the analyzed data set. Only 11 different haplogroups were found with 18 SNPs analyses, and E1b1b1 and R1 haplogroups seem representative of two different meta-populations, Albanians/North Africans and Italians/Indo-Pakistanis; in fact, R1 was the most frequent haplogroup both in Italians (60.80%) and Indo-Pakistanis (38.60%), while E1b1b1 was the most frequent haplogroup both in Albanians (32.50%) and North Africans (57.10%). J2 haplogroup was almost equally represented in each population (ranging from 6.54%, in Italians to 16.87% in Albanians). Other haplogroups were not significantly represented in the data set. Meta-populations described above are concordant with already described European populations (19,20).

Haplogroup prediction starting from haplotype is theoretically possible under certain conditions (21,22) and if Y-STRs are routinely tested in forensic laboratories, they could be greatly useful for forensic investigations. No strong correlations were observed in the data set between STRs-specific alleles and haplogroups. Haplogroup prediction from the haplotype was not calculated in this study since a simple Bayes theorem calculation would be strongly affected by a sampling error due to the small data set.

In conclusion, SNPs analysis seems to be a powerful tool to infer the ethnic origin of an unknown sample but the number of samples for each of these four populations needs to be increased for a better resolution. Our results showed a low discrimination considering the haplotype and the haplogroup independently. Otherwise, the combination of the two systems enabled good discrimination between Italian men from northern Italy and men belonging to other three ethnic groups, which has potential usefulness in crime scene investigations. Further autochthonous population studies are needed to highlight the most informative loci.
